# KLHL38 involvement in non-small cell lung cancer progression via activation of the Akt signaling pathway

**DOI:** 10.1038/s41419-021-03835-0

**Published:** 2021-05-28

**Authors:** Yitong Xu, Chenglong Wang, Xizi Jiang, Yao Zhang, Hongbo Su, Jun Jiang, Hongjiu Ren, Xueshan Qiu

**Affiliations:** 1 Department of Pathology, The First Affiliated Hospital, China Medical University, 110000 Shenyang, China; 2 Department of Pain Medicine, The First Affiliated Hospital, China Medical University, 110000 Shenyang, China; 3 Department of Pathology, College of Basic Medical Sciences, China Medical University, 110000 Shenyang, China; 4 Department of Pathology, The First Bethune Hospital of Jilin University, 130000 Changchun, Jilin China

**Keywords:** Lung cancer, Oncogenesis

## Abstract

Lung cancer is the leading cause of cancer-related death worldwide. KLHL38 has been reported to be upregulated during diapause but downregulated after androgen treatment during the reversal of androgen-dependent skeletal muscle atrophy. This study aimed to clarify the role of KLHL38 in non-small cell lung cancer (NSCLC). KLHL38 expression was evaluated in tumor and adjacent normal tissues from 241 patients with NSCLC using immunohistochemistry and real-time PCR, and its association with clinicopathological parameters was analyzed. KLHL38 levels positively correlated with tumor size, lymph node metastasis, and pathological tumor-node-metastasis stage (all *P* < 0.001). In NSCLC cell lines, *KLHL38* overexpression promoted PTEN ubiquitination, thereby activating Akt signaling. It also promoted cell proliferation, migration, and invasion by upregulating the expression of genes encoding cyclin D1, cyclin B, c-myc, RhoA, and MMP9, while downregulating the expression of p21 and E-cadherin. In vivo experiments in nude mice further confirmed that KLHL38 promotes NSCLC progression through Akt signaling pathway activation. Together, these results indicate that KLHL38 is a valuable candidate prognostic biomarker and potential therapeutic target for NSCLC.

## Introduction

Lung cancer is a major global public health concern. Lung cancer accounts for 11.4% of all new cancer cases diagnosed worldwide and is the second most common cancer overall^1^. Lung cancer remains the leading cause of cancer-related death, 1.8 million people die annually due to this malignancy. Approximately 85–90% of lung cancer cases are diagnosed as non-small cell lung cancer (NSCLC)^2^. The 5-year survival rates for NSCLC vary within 4–17%, depending on the stage and region. Whenever possible, surgery is the best treatment strategy for NSCLC; however, patients may have a local or systemic recurrence and ultimately succumb to the disease. The prognosis of patients with NSCLC mainly correlates with tumor metastasis, which is associated with the transcriptional regulation of critical genes^3,4^, therefore, it is necessary to identify pro-metastatic genes and investigate their effects in detail.

The PI3K/Akt signaling pathway is commonly dysregulated in various cancers, including lung cancer^5^, and plays a key role in cancer development. Phosphorylated (p)-Akt, the activated form of Akt, is highly expressed in several human cancers and is associated with survival rate in some cancer types. Akt is commonly phosphorylated at two sites, Ser473 and Thr308, which result in full or partial activation, respectively. PI3K-related kinase family members, including DNA-PK, can phosphorylate Akt at Ser473. In contrast, Akt can be dephosphorylated by protein phosphatase 2A and PH domain leucine-rich repeat inclusion protein phosphatase. The tumor suppressor phosphatase and tensin homologous protein (PTEN) is also a major negative regulator of Akt via PIP3 dephosphorylation^6–8^.

PTEN is a powerful tumor suppressor, even a partial reduction in its expression leads to increased cancer susceptibility^9^. Several studies have reported a consistently low *PTEN* mutation rate (~2–7%) in lung cancer^10,11^. In contrast, the rate of PTEN loss is reported to be high in lung cancer. PTEN-negative lung cancer patients with lymph node metastasis, who are usually smokers, have the worst survival rates^12^. The development of tyrosine kinase inhibitors targeting epidermal growth factor receptor (EGFR), such as gefitinib, afatinib, and erlotinib, has been a breakthrough for treating patients with NSCLC who carry *EGFR* mutations. However, the emergence of resistance to EGFR tyrosine kinase inhibitors is almost inevitable in these oncogene-affected tumors, among which *PTEN* deletion and low PTEN levels are strong and independent predictors of poor outcomes^13,14^. The most critical function of PTEN is to negatively regulate the PI3K/Akt pathway, thereby inhibiting uncontrolled cell proliferation, growth, and migration. However, the lack of genetic alterations, such as mutations and deletions, in *PTEN* in lung cancer does not correlate with the observed loss of protein, suggesting a possible role for post-transcriptional regulation^5^.

KLHL38 is a member of the KLHL family and contains a BTB domain, a back domain, and six kelch domains^15^.KLHL38 expression is reportedly upregulated during diapause and downregulated after androgen treatment during the reversal of androgen-dependent skeletal muscle atrophy, in which the Akt signaling pathway is involved^16^. Using database mining (http://www.proteinatlas.org/) and analysis, KLHL38 may be a potential indicator for prognosis of lung adenocarcinoma and squamous cell carcinoma. Moreover, database searches have also suggested that KLHL38 could be involved in NSCLC via the Akt signaling pathway.

According to the Kaplan–Meier database (http://kmplot.com/), KLHL38 may act as a tumor promoter in NSCLC. However, the role of KLHL38 in lung cancer is currently unclear. No data are available regarding the expression pattern and clinical significance of KLHL38 in NSCLC. Thus, this study aimed to determine the role of KLHL38 in human NSCLC cells.

## Materials and methods

### Patients and specimens

A total of 241 tissue specimens from patients with NSCLC, surgically treated between 2013 and 2017, were randomly selected from the Pathology Archive of the First Affiliated Hospital of China Medical University. Among the 241 samples, 43 and 16 samples of NSCLC tumor and paracancerous lung tissue from surgical procedures in 2015, respectively, were randomly selected for real-time PCR and western blot analysis. None of the 241 patients who participated in the pathological correlation analysis received preoperative chemotherapy or radiotherapy. Clinicopathological information was obtained from the clinical records of the patients. This study was approved by the Medical Research Ethics Committee of China Medical University, and informed consent was obtained from all patients (AF-SOP007-1.1-01).

### Immunohistochemistry

The instruments and methods used for immunohistochemical analyses were described previously^17^. Immunostaining with an anti-KLHL38 rabbit polyclonal antibody (1:100; Sigma-Aldrich, St. Louis, MO, USA) was performed at 4 °C for 18 h. The signal was visualized using Elivision Super HRP (Mouse/Rabbit) IHC Kits (Maixin-Bio, Fuzhou, China) and 3,3′-diaminobenzidine (Maixin-Bio); nuclei were counterstained with hematoxylin. The sections were dehydrated in ethanol before mounting.

Single-blinded scoring of the KLHL38 staining intensity was performed as follows: 0 (no staining), 1 (weak staining), 2 (moderate staining), and 3 (high staining). Scores for the percentage of cells stained were assigned as 0 (0%), 1 (1–30%), 2 (31–70%), and 3 (71–100%). The two scores for each tumor sample were multiplied to obtain a final score ranging from 0 to 9, with scores ≥3 being considered KLHL38-positive and scores <3 being considered KLHL38-negative.

### Quantitative real-time polymerase chain reaction (qRT-PCR)

Total RNA was extracted from 43 pairs of fresh clinical lung tumor tissues with matched adjacent normal lung tissues using TRIzol reagent (ThermoFisher Scientific, Waltham, MA, USA). RNA in the aqueous phase was precipitated using trichloromethane, isopropanol, and 70% ethanol. Reverse transcription was performed using PrimeScriptRT Reagent Kits with gDNA Eraser (Takara Bio, Beijing, China). qRT-PCR was performed on a 7900HT Fast Real-Time PCR System (Applied Biosystems, Foster City, CA, USA) using SYBR Green PCR Master Mix (Takara Bio) in 20 μl final volume with the following thermocycling conditions: 95 °C for 30 s, followed by 45 cycles at 95 °C for 5 s, then 60 °C for 30 s. A melting curve was generated to confirm the specificity of amplification. Relative gene expression was calculated using the 2^−ΔΔCt^ method with *ACTB* as the internal control. The primer sequences used were as follows: *KLHL38* (forward) 5′–GGCCCTCATGGTTTGGATCA–3′ and (reverse) 5′–ATCGTTGGCGATGAAGTGGT–3′; *ACTB* (forward) 5′–ATAGCACAGCCTGGATAGCAACGTAC–3′ and (reverse) 5′–CACCTTCTACAATGAGCTGCGTGTG–3′; *PTEN* (forward) 5′–CCCAGTTTGTGGTCTGCCAGC–3′ and (reverse) 5′–ATGAGCTTGTCCTCCCGCCG–3′. Dissolution curves ensured the validity of amplification. All experiments were performed in triplicate.

### Cell culture and treatment

The lung cancer cell lines A549, H1299, H460, H661, and SK-MES-1 were purchased from the Cell Bank of the China Academy of Sciences (Shanghai, China), and normal bronchial epithelial HBE cells were obtained from the American Type Culture Collection (Manassas, VA, USA). A549, H1299, H460, and H661 cells were cultured in RPMI 1640 medium (Gibco, Waltham, MA, USA), SK-MES-1 cells were cultured in minimal essential medium (Gibco) containing 1.5 g/l NaHCO_3_ and 0.11 g/l sodium pyruvate, and HBE cells were cultured in Dulbecco’s modified Eagle medium (Gibco) containing 1.5 g/l NaHCO_3_. All media were supplemented with 10% fetal bovine serum (CLARK Bioscience, Richmond, VA, USA). The cells were maintained in a 5% CO_2_ humidified incubator at 37 °C.

Cells were transfected using Lipofectamine 3000 (Invitrogen, Waltham, MA, USA) according to the manufacturer’s instructions. For *KLHL38* knockdown experiments, cells were transfected with *KLHL38*-specific siRNA or scrambled control siRNA (GenePharma, Shanghai, China) for 48 h. For *KLHL38* overexpression, cells were transfected with a pCMV6-myc-DDK-*KLHL38* plasmid or the corresponding pCMV6-myc-DDKempty vector.

To inhibit PTEN signaling, cells were treated with the PTEN inhibitor HY-128693 (10 µM, SelleckChem, Houston, TX, USA) in DMSO (Sigma-Aldrich). Cells were treated with HY-128693 or an equal volume of DMSO 6 h after transfection and incubated for 36 h.

### Immunocytochemistry

Lung cancer cell lines were cultured in 24-well plates for 24 h, fixed using 2% paraformaldehyde for 15 min, blocked with 5% bovine serum albumin for 2 h, and incubated with an anti-KLHL38 antibody (1:100) for 18 h, followed by the addition of a fluorescein isothiocyanate-conjugated secondary antibody (Beyotime Biosciences, Shanghai, China) for 2 h; nuclei were counterstained with DAPI (Beyotime) for 15 min. Cell images were captured using an Olympus FV3000 laser-scanning confocal microscope (Olympus, Tokyo, Japan). The exposure time varied with the intensity of the fluorescent signal.

### Western blotting

The expression of proteins directly influencing cell migration and invasion^18–20^ and those involved in cell proliferation and cell cycle progression^21,22^ was analyzed using western blotting. Total protein from cells and tumor tissues was extracted with lysis buffer and quantified using the Bradford method. Proteins (80 µg/lane) were separated using 10% sodium dodecyl sulfate-polyacrylamide gel electrophoresis (SDS-PAGE), transferred onto polyvinylidene fluoride membranes (Millipore, Billerica, MA, USA), and incubated overnight at 4 °C with the appropriate primary antibodies (Table 1), followed by addition of a horseradish peroxidase-conjugated anti-mouse/rabbit IgG secondary antibody at 37 °C for 2 h. Immune reactivity was detected using enhanced chemiluminescence (ThermoFisher Scientific) on a BioImaging System (UVP Inc., Upland, CA, USA). Relative protein levels were calculated after normalization to GAPDH, which was used as a loading control.

**Table 1:** List of antibodies used for western blotting.

Target	Source	Catalog number	Host	Dilution
KLHL38	Sigma-Aldrich	HPA030464	Rabbit	1:500
GAPDH	Beyotime	AF0006	Mouse	1:1000
β-Actin	Beyotime	AA128	Mouse	1:1000
RhoA	Cell Signaling Technology	2117	Rabbit	1:500
MMP9	Cell Signaling Technology	13667	Rabbit	1:500
Cyclin D1	Cell Signaling Technology	2978	Rabbit	1:500
CyclinB1	Cell Signaling Technology	12231	Rabbit	1:500
p21	Cell Signaling Technology	2947	Rabbit	1:1000
c-Myc	Cell Signaling Technology	13987	Rabbit	1:500
E-Cadherin	Cell Signaling Technology	3195	Rabbit	1:500
PTEN	Cell Signaling Technology	9188	Rabbit	1:500
Akt	Cell Signaling Technology	9272	Rabbit	1:500
P-Akt (Ser473)	Cell Signaling Technology	4060	Rabbit	1:500
Anti-mouse IgG	Cell Signaling Technology	7076	Horse	1:1000
Anti-rabbit IgG	Cell Signaling Technology	7074	Goat	1:1000

### Gelatin zymograph assay

Cells were seeded onto 6-well plates and incubated in a serum-free medium for 24 h. Supernatants were then collected and the protein concentration was quantified. The cell supernatants were mixed with 4× SDS loading buffer followed by electrophoresis on 10% SDS-PAGE gels containing 1% gelatin at 4 °C. The gel was washed with eluent buffer (2.5% Triton X-100, 50 mmol/l Tris-HCl, 5 mmol/l CaCl_2_, pH 7.6) for 15 min four times, and rinsed (50 mmol/l Tris-HCl, 5 mmol/l CaCl_2_, pH 7.6) for 20 min twice. The gels were then incubated in reaction buffer (50 mmol/l Tris-HCl, 5 mmol/l CaCl_2_, 0.02% Brij-35, pH 7.6) at 37 °C for 48 h, stained with 0.05% Coomassie brilliant blue for 3 h, and destained with buffer containing 30% methanol and 10% acetic acid for 2 h before each band was photographed.

### Co-immunoprecipitation assays

Cells were plated in 10-cm dishes. Cell monolayers were lysed using NP40 and lysates were centrifuged at 4000×*g* for 20 min. Then, 60 μl of Protein A/G Sepharose (P2012; Beyotime Biosciences) was added to the lysate, blocked for 2 h, then centrifuged at 2000 rpm to remove the magnetic beads. The remaining protein lysate was divided into two parts, and 5 µg target antibody and anti-mouse/rabbit IgG were added to each part and incubated overnight at 4 °C while shaking in a chromatography cabinet. The next day, 25 μl agarose A/G magnetic beads were added to each tube and incubated at 4 °C for 6 h; the cell lysate was then washed with lysis buffer and the tubes were heated in boiling water for 10 min. The samples were then used for western blotting.

### Ubiquitination assays and immunoprecipitation

The ubiquitination and immunoprecipitation assays were performed as described previously^23,24^.

### Cell proliferation and colony formation assays

Cell proliferation and colony formation assays were performed as described previously^17^.

### Cell migration and invasion analysis

Cell migration and invasion assays using the A549 and H1299 cell lines were performed as described previously^17^. Assays with SK-MES-1 cells were performed using the same experimental protocol, but the number of inoculated cells was 8 × 10^4^ cells/100 μl culture medium.

### Tumor formation in nude mice

Nude mice used in this study were handled following the experimental animal ethics guidelines issued by the China Medical University.

Four-week-old female BALB/c nude mice were purchased from Slac (Shanghai, China) and were maintained in a laminar-flow cabinet under specific pathogen-free conditions for one week before use. Each mouse was inoculated subcutaneously in the right axilla with 1 × 10^7^ tumor cells (*KLHL38*-transfected A549 and SK-MES-1 cells, or the corresponding vector-transfected control cells) in 0.2 ml sterile phosphate-buffered saline (PBS) or in the tail vein with 1 × 10^6^ tumor cells in 0.1 ml PBS. Four or 8 weeks after axilla and tail vein inoculation, respectively, the mice were euthanized, and necropsies were performed to examine tumor growth and dissemination. A portion of tissue from the tumor and each organ was fixed in 4% formaldehyde (Sigma-Aldrich) and embedded in paraffin. Serial 4 µm-thick sections were cut and stained with hematoxylin and eosin and examined microscopically. The animal care and experimental protocols were approved by the Medical Research Ethics Committee of the China Medical University (CMU2020406).

### Statistical analysis

SPSS v16.0 software (SPSS Inc., Chicago, IL, USA) was used for data analysis. Correlations between KLHL38 expression and clinicopathological features were determined using the chi-square test, and differences between cell groups were determined using paired *t*-tests. Results with two-sided *P* < 0.05 were considered statistically significant.

## Results

### KLHL38 is overexpressed in NSCLC and correlates with clinicopathological parameters

**Fig. 1:**
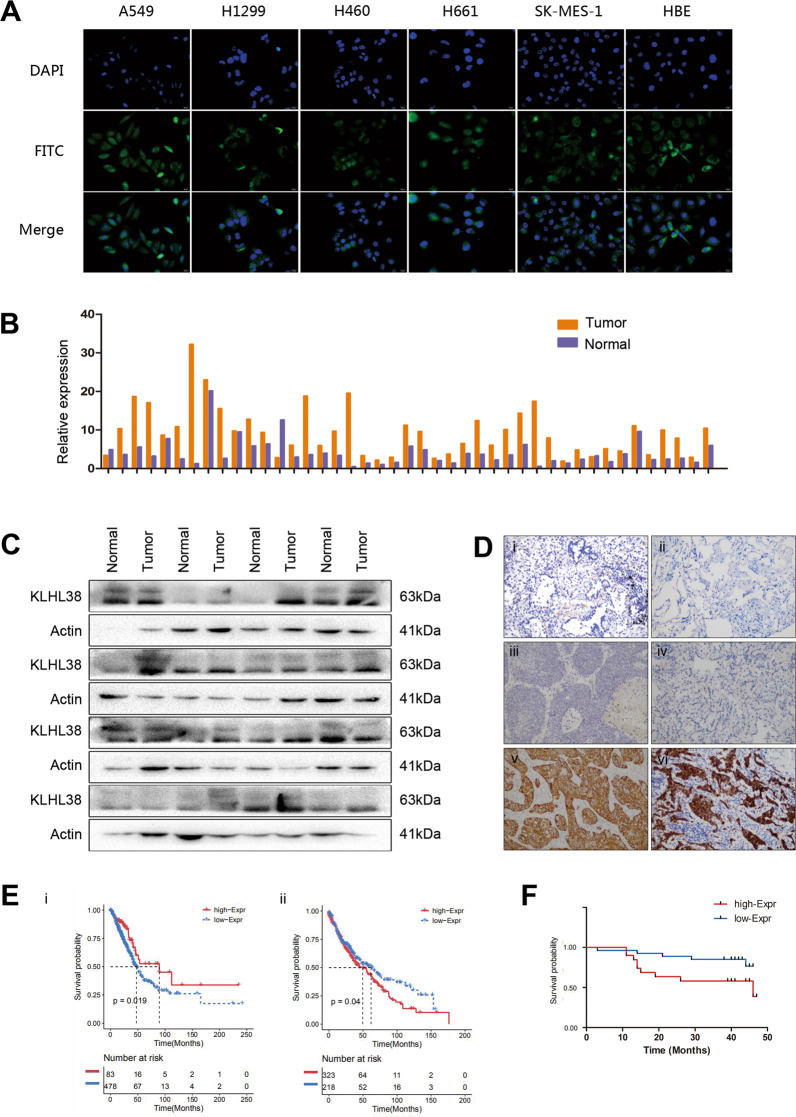
Expression of KLHL38 in non-small cell lung cancer (NSCLC) cells is associated with poor clinical prognosis. **A** Immunofluorescence was performed to detect KLHL38 localization in NSCLC cell lines and human normal bronchial epithelial cells. **B** Relative expression of *KLHL38* in 43 paired NSCLC samples (orange bars) and paracancerous lung tissue (purple bars). **C** KLHL38 levels in 14 lung cancer tissues and matched normal tissues. **D** KLHL38 levels in alveolar (i) and normal bronchial epithelial cells (ii), well-differentiated squamous cell carcinoma (iii) and adenocarcinoma (iv), and poorly differentiated squamous cell carcinoma (v) and adenocarcinoma (vi) using immunohistochemistry. Magnification: ×200. **E** Survival analysis of NSCLC patients with high and low *KLHL38* expression in lung adenocarcinoma (i) and lung squamous cell carcinoma (ii) based on TCGA data. **F** Survival of NSCLC patients with high and low KLHL38 expression analyzed based on the results of the present study.

Immunocytochemical analysis indicated that KLHL38 was localized in both the cytoplasm and nuclei of A549, H1299, H460, H661, and SK-MES-1 cells and normal bronchial epithelial HBE cells (Fig. 1A).

Overall, 43 matched pairs of clinical lung cancer and normal tissue samples were selected for RNA extraction, and *KLHL38* levels were measured. *KLHL38* levels were significantly higher in cancers than in matched normal tissues (single sample paired *t*-test, *P* = 0.000) (Fig. 1B).

Western blotting confirmed that KLHL38 levels were higher in 14 of the 16 lung cancer tissues analyzed than in the matched normal tissues (Fig. 1C). Moreover, immunohistochemical results showed that KLHL38 was overexpressed in clinical lung cancer tissues, whereas in normal bronchi and alveoli, KLHL38 was minimally expressed. Correlation (cross table) analysis with clinicopathological factors revealed that KLHL38 expression did not correlate with the tumor histological type (*P* = 0.198), degree of differentiation (*P* = 0.170), patient age (*P* = 0.699), or sex (*P* = 0.276), but did correlate positively with tumor size (*P* = 0.000), lymph node metastasis (*P* = 0.000), and p-TNM stage (*P* = 0.000). Statistical analyses were performed for all cases, as well as separately for adenocarcinoma and squamous cell carcinoma (Table 2). KLHL38 differential expression results are shown in Fig. 1D. Analysis of The Cancer Genome Atlas (TCGA) database also showed a differential correlation of KLHL38 expression with patient prognoses (Fig. 1E). Of note, statistical analysis of clinical samples suggested that high expression of KLHL38 was associated with poor prognosis (Fig. 1F), whereas statistical analysis of TCGA data for lung adenocarcinoma (Fig. 1E-i) and lung squamous carcinoma (Fig. 1E-ii) suggested the opposite.

**Table 2:** Association of KLHL38 expression with clinical and pathological characteristics of patients.

	NSCLC			*P*-value	Lung adenocarcinoma			*P*-value	SCC			*P*-value
Clinicopathological characteristics	Samples (*N*)	KLHL38-positive	KLHL38-negative		Samples (*N*)	KLHL38-positive	KLHL38-negative		Samples (*N*)	KLHL38-positive	KLHL38-negative	
Age (years)												
≤60	129	64	65		68	31	37		61	33	28	
>60	112	59	53	0.699	54	26	28	0.856	58	33	25	0.854
Gender												
Male	160	86	74		62	33	29		98	53	45	
Female	81	37	44	0.276	60	24	36	0.152	21	13	8	0.631
Differentiation												
Well-moderate	162	88	74		48	20	28		31	15	16	
Poor	79	35	44	0.170	74	37	37	0.458	88	51	37	0.404
Tumor size (cm)												
≤3	134	47	87		80	31	49		54	16	38	
>3	107	31	76	0.000	42	26	16	0.022	65	50	15	0.000
Lymph node metastasis												
Negative	142	51	91		72	24	48		70	27	43	
Positive	99	72	27	0.000	50	33	17	0.000	49	39	10	0.000
TNM stage												
I	102	23	79		55	15	40		47	8	39	
II–III	139	100	39	0.000	67	42	25	0.000	72	58	14	0.000
Histological type												
SCC	119	66	53									
Adenocarcinoma	122	57	65	0.198								

*NSCLC* non-small cell lung carcinoma, *SCC* squamous cell carcinoma, *TNM* Eighth Edition of tumor-node-metastasis classification.

### KLHL38 promotes the migration and invasion of lung cancer cells

**Fig. 2:**
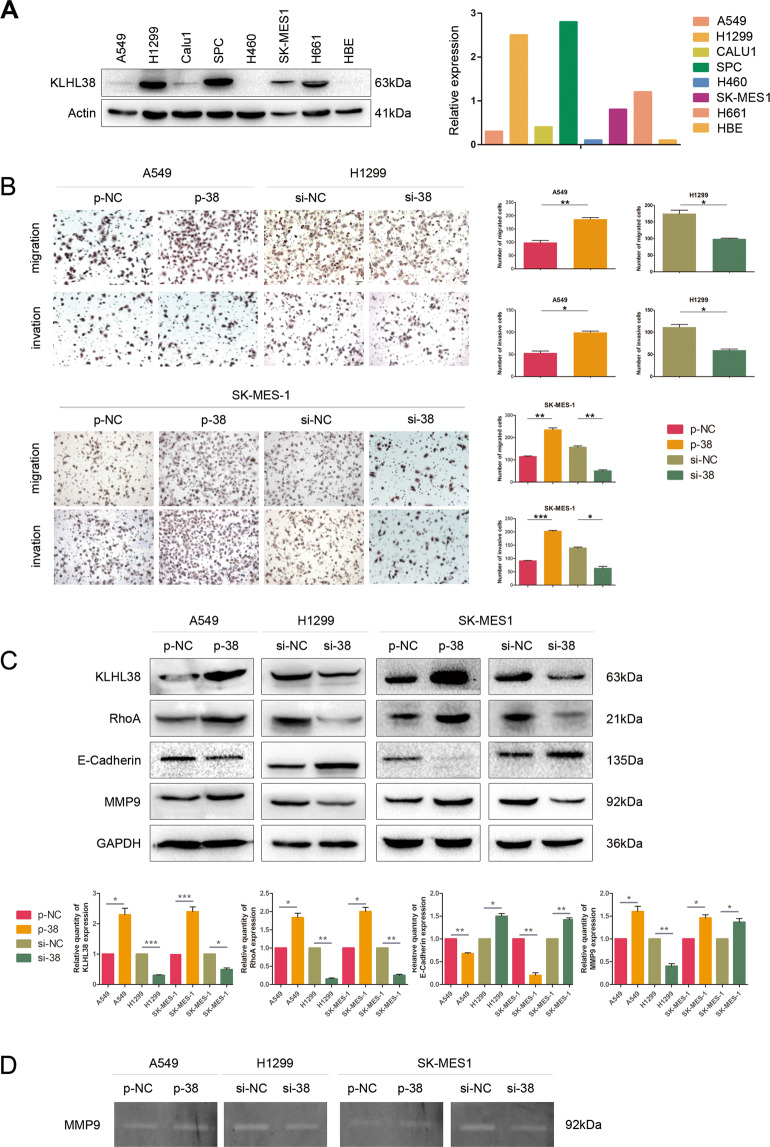
Effect of KLHL38 expression on the migration and invasion of non-small cell lung cancer (NSCLC) cells. **A** KLHL38 levels in HBE cells and seven NSCLC cell lines determined by western blotting**. B** Cell migration evaluated by the transwell migration assay; cells that migrated to the lower chamber were stained with hematoxylin and counted. *P < 0.05; **P < 0.01. **C** Expression of KLHL38 and cell migration- and invasion-related proteins in A549, H1299, and SK-MES-1 cells. Relative quantification of western blot data was based on densitometry analysis. p-NC, cells transfected with pCMV6-myc-DDK; p-38, cells transfected with pCMV6-myc-DDK-*KLHL38*; si-NC, cells transfected with scrambled control siRNA; si-38, cells transfected with *KLHL38*-specific siRNA. **D** Gelatin zymograph assay showing the relationship between KLHL38 and secreted active MMP9.All experiments were repeated three times, error bars as s.d.

As KLHL38 expression was correlated with lymph node metastasis, we speculated that KLHL38 could be related to the migration and invasion of lung cancer cells. To study the role of KLHL38 in NSCLC, its expression was first evaluated in several cancer cell lines (Fig. 2A), of which A549, H1299, and SK-MES-1 cells had low, high, and intermediate levels of KLHL38, respectively; hence, these cell lines were selected for studying the effects of KLHL38 expression. KLHL38 was knocked down using siRNA transfection and upregulated by transfecting with a pCMV6-myc-DDK-*KLHL38* plasmid. In both A549 and H1299 adenocarcinoma cells and in SK-MES-1 squamous cells, Moreover, KLHL38 expression was significantly associated with the promotion of migration and invasion (Fig. 2B). Variations in KLHL38 levels also affected the expression of proteins involved in migration and invasion. Overexpression of *KLHL38* resulted in upregulation of RhoA and MMP9 and downregulation of E-cadherin. These effects were reversed by *KLHL38* knockdown (Fig. 2C). Gelatinase analysis showed that KLHL38 could positively regulate the secretion of active MMP9 (Fig. 2D). Collectively, these findings demonstrate that KLHL38 promotes the migration and invasion of lung cancer cells.

### KLHL38 promotes the proliferation of lung cancer cells

**Fig. 3:**
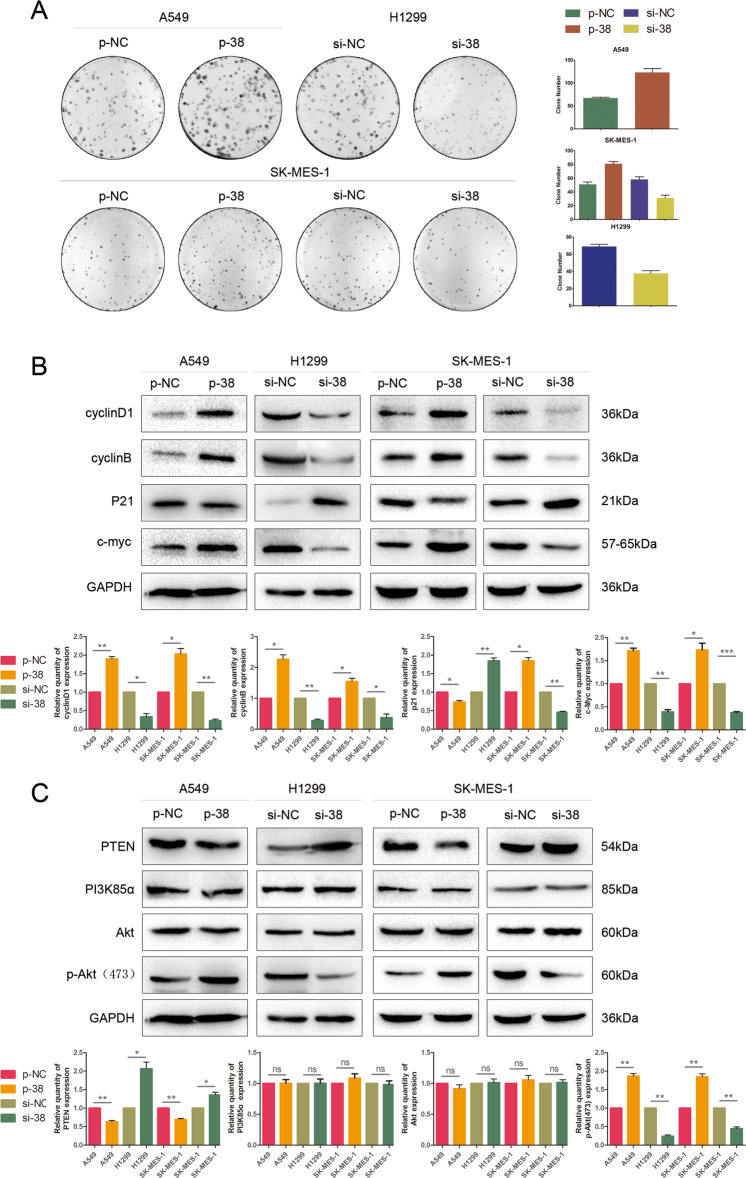
Effect of KLHL38 expression on colony formation and Akt signaling. **A** Colony formation assay. A549: pCMV6-myc-DDK-*KLHL38* (p-38) vs. pCMV6-myc-DDK (p-NC), *P* = 0.0278. H1299: *KLHL38*-specific siRNA (si-38)vs. scrambled control siRNA (si-NC), *P* = 0.0189; SK-MES-1: p-38 vs. p-NC, P = 0.0391, si-38 vs. si-NC, *P* = 0.0318. **B** Changes in the expression of proliferation-related proteins in A549, H1299, and SK-MES-1 cells. **C** Changes in Akt signaling in A549, H1299, and SK-MES-1 cells. p-NC, cells transfected with pcmv6-myc-DDK; p-38, cells transfected with pCMV6-myc-DDK-*KLHL38*; si-NC, cells transfected with scrambled control siRNA; si-38, cells transfected with *KLHL38*-specific siRNA. All experiments were repeated three times, error bars as s.d.

The effect of KLHL38 expression on tumor size was evaluated as a reflection of cell proliferation. In A549 and SK-MES-1 cells, KLHL38 upregulation promoted clone formation, whereas, in H1299 and SK-MES-1 cells, KLHL38 downregulation had the opposite effect (Fig. 3A). Analysis of proteins involved in cell proliferation indicated that the levels of cyclin D1, cyclin B, and c-myc were upregulated, whereas p21 was downregulated upon *KLHL38* overexpression. KLHL38 downregulation had the opposite effects (Fig. 3B).

### KLHL38 promotes NSCLC progression via Akt signaling pathway activation

As cell proliferation-related proteins are targeted by the Akt signaling pathway, we next investigated if the potential relationship with KLHL38 existed. PTEN/PI3K/AKT constitutes an important pathway regulating the signaling of multiple biological processes such as apoptosis, metabolism, cell proliferation, and cell growth^6^. The Akt inhibitor AKT VIII (MCE, Monmouth Junction, NJ, USA) and si-PTEN were used separately to verify the function of PTEN/PI3K/Akt/phospho-Akt in this pathway. Downregulation of PTEN caused an upregulation of p-AKT (Supplementary Fig. S1A), while treatment with Akt VIII caused decreases in Akt and p-Akt expression, but no effect was observed on PI3K and PTEN expression (Supplementary Fig. S1B). PTEN can therefore inhibit the Akt pathway through inhibition of Akt phosphorylation. PTEN was found to be significantly upregulated in the three analyzed cell lines, whereas p-Akt (Ser473) was downregulated after *KLHL38* knockdown; KLHL38 upregulation had the opposite effect (Fig. 3C).

**Fig. 4:**
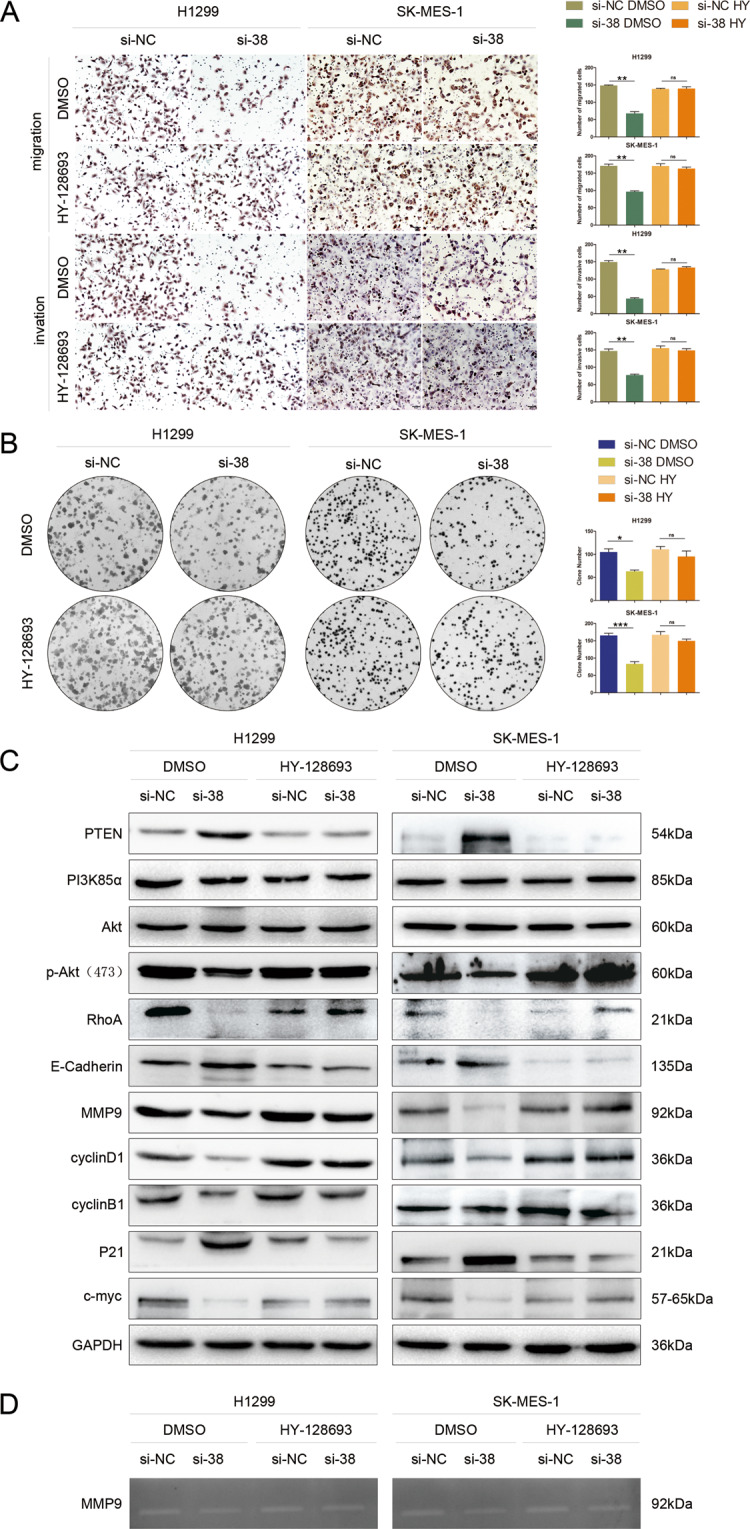
Effect of KLHL38 expression with or without PTEN inhibition on non-small cell lung cancer (NSCLC) cells. **A** Migration, invasion, and **B** colony formation assays of H1299 and SK-MES-1 cells transfected with a *KLHL38*-specific siRNA were reversed upon incubation with the PTEN inhibitor HY-128693, compared to DMSO treated cells. **C** Expression of proteins involved in cell proliferation, migration, invasion, and the Akt signaling in A549, H1299, and SK-MES-1 cells transfected with a *KLHL38*-specific siRNA upon treatment with DMSO or the PTEN inhibitor HY-128693. si-NC, cells transfected with scrambled control siRNA; si-38, cells transfected with *KLHL38*-specific siRNA. **D** Gelatin zymograph assay showing the changes of secreted active MMP9. All experiments were repeated three times, error bars as s.d.

To investigate whether KLHL38 could promote Akt activation by reducing PTEN levels, the PTEN inhibitor HY-128693 was used. In H1299 and SK-MES-1 cells transfected with *KLHL38*-siRNA, HY-128693 partially reduced the induction of cell migration, invasion, and proliferation (Fig. 4A, B), reversed the effects of *KLHL38* knockdown on protein expression (Fig. 4C), and induced secretion of active MMP9 (Fig. 4D). These results suggest that *KLHL38* overexpression activates the Akt pathway via PTEN inhibition.

### KLHL38 interacts with PTEN and promotes its ubiquitination and degradation

**Fig. 5:**
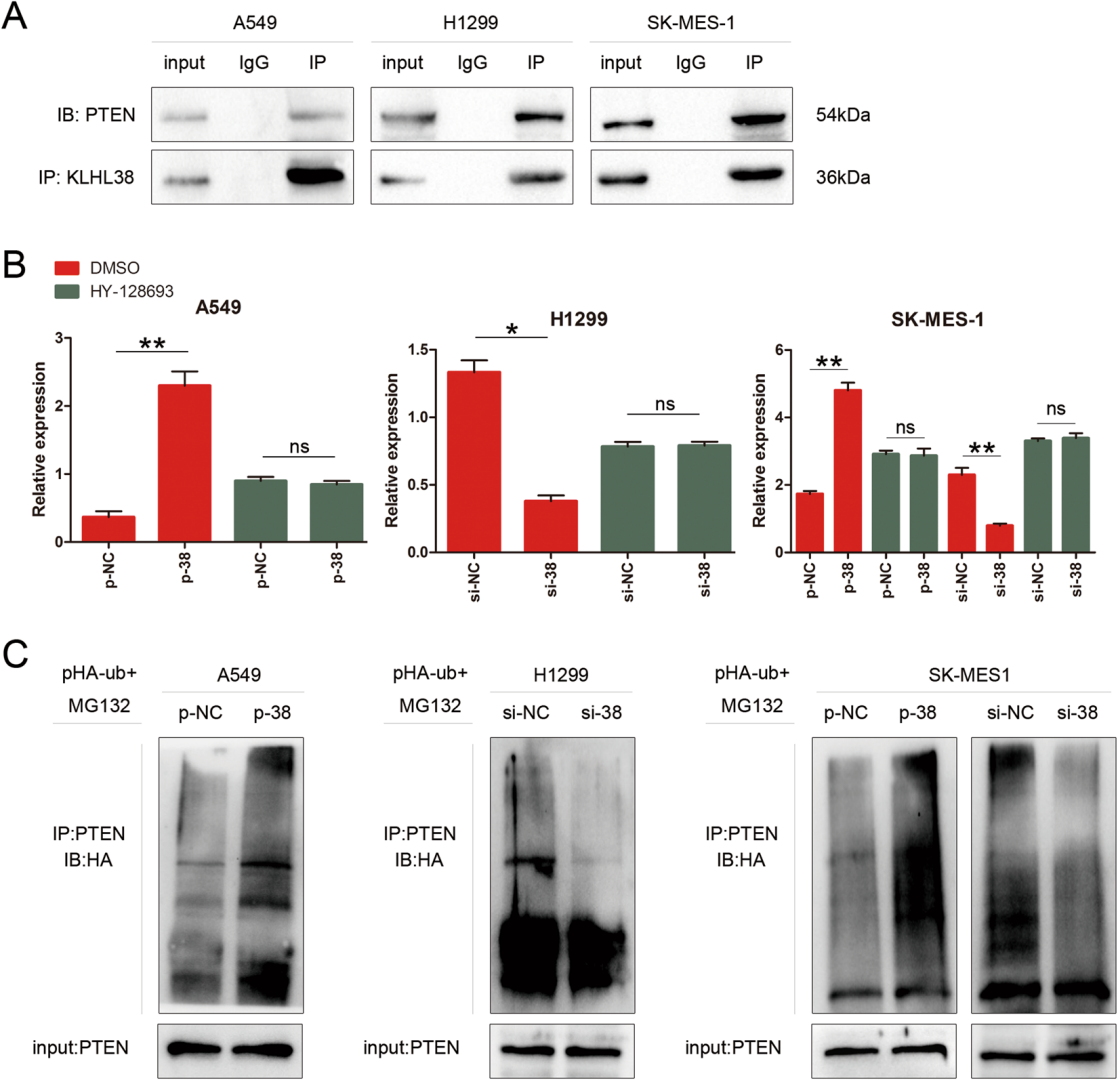
Relationship between KLHL38 and PTEN. **A** Interactions between KLHL38 and PTEN in A549, H1299, and SK-MES-1 cells measured by co-immunoprecipitation. **B** *PTEN* levels in A549, H1299, and SK-MES-1 cells upon up- or downregulation of *KLHL38* expression. **C** A549, H1299, and SK-MES-1 cells were transfected with a *KLHL38*-specific siRNA or pCMV6-myc-DDK-*KLHL38* along with HA-ubiquitin (Ub). Levels of PTEN ubiquitination were evaluated by immunoprecipitation using an anti-PTEN antibody, followed by anti-HA immunoblotting. p-NC, cells transfected with pCMV6-myc-DDK; p-38, cells transfected with pCMV6-myc-DDK-*KLHL38*; si-NC, cells transfected with scrambled control siRNA; si-38, cells transfected with *KLHL38*-specific siRNA. All experiments were repeated three times, error bars as s.d.

Results of co-immunoprecipitation experiments revealed that KLHL38 directly interacts with PTEN in lung cancer cells (Fig. 5A). However, the qRT-PCR analysis indicated that *PTEN* expression was unchanged regardless of *KLHL38* expression in lung cancer cell lines (Fig. 5B), indicating that the interaction between KLHL38 and PTEN may occur at the protein rather than mRNA level. Considering that the members of the KLHL protein family possess ubiquitination ability, KLHL38 could also contribute to the activation of the Akt signaling pathway by enhancing PTEN ubiquitination. Therefore, HA-ubiquitin was transfected into cells, along with siRNA or plasmids, which were then treated with the 26S proteasome inhibitor MG132. Next, PTEN ubiquitination levels were evaluated by immunoprecipitation with an anti-PTEN antibody, followed by anti-HA immunoblotting. The results revealed a positive correlation between KLHL38 expression and PTEN ubiquitination (Fig. 5C), suggesting that KLHL38 activates the Akt pathway by facilitating ubiquitin-dependent degradation of PTEN.

### KLHL38 promotes cell proliferation, migration, and invasion in vivo

**Fig. 6:**
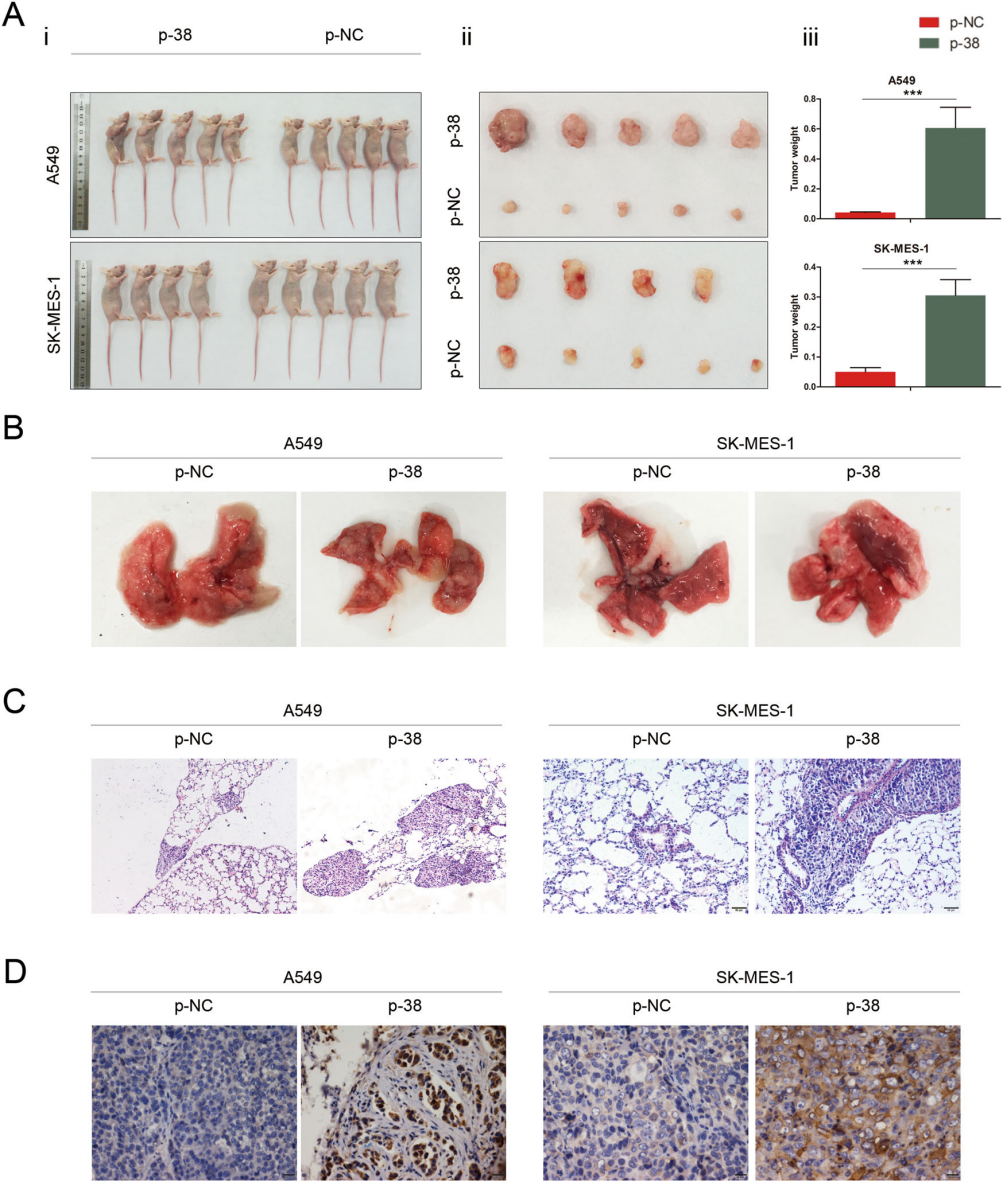
KLHL38 influences cell proliferation, migration, and invasion in vivo. **A** Xenograft tumor volumes were positively affected by KLHL38, i. Comparison of gross photos of nude mice; ii. Gross picture of subcutaneous tumor; iii. Histograms of subcutaneous tumorigenesis. **B** Mice injected with lung cancer cells overexpressing KLHL38 showed enhanced pulmonary metastases compared to the control group. **C** Hematoxylin and eosin staining of metastatic tumor tissues. Magnification: ×200**. D** KLHL38 staining of metastatic tumors by immunohistochemistry. Magnification: ×400. p-NC, cells transfected with pCMV6-myc-DDK; p-38, cells transfected with pCMV6-myc-DDK-*KLHL38*. Error bars as s.d.

A549 and SK-MES-1 cells were used to generate stably transfected cell lines using G418 and the relevant plasmids. Changes in KLHL38 expression in these cells positively affected the xenograft tumor volume (Fig. 6A) and weight (Fig. 6B). The lung metastatic rate (5/5 vs. 1/5 and 4/5 vs. 1/4 for A549 and SK-MES-1, respectively) and the number of lung metastatic nodules in mice transplanted with A549-KLHL38(+) or SK-MES-1-KLHL38(+) cells were significantly greater than those in the control group (Fig. 6C). These in vivo results further confirm that *KLHL38* overexpression enhances the proliferation, migration, and invasion of NSCLC cells.

## Discussion

This study demonstrated that KLHL38 localizes in the cytoplasm and nucleus, and that its expression level correlates with a range of clinicopathological parameters in NSCLC, including tumor size, lymph node metastasis, and pathological tumor-node-metastasis stage. We used a 3 cm cut-off value to determine T1 and T2 in the p-TNM stage of lung cancer. Our statistical study found that the cut-off value for KLHL38 and tumor size was exactly the referred value, which also suggests clinical significance. In vitro and in vivo experiments using NSCLC cell lines confirmed that KLHL38 promotes their proliferation, migration, and invasion. In vitro experiments using the adenocarcinoma cell lines A549 and H1299 and the squamous cell line, SK-MES-1 demonstrated that KLHL38 promotes migration and invasion by upregulating RhoA and MMP9 and downregulating E-cadherin. To promote proliferation, KLHL38 also positively regulates cyclin D1, cyclin B, and c-myc, whereas it negatively regulates p21.

The role of the Akt pathway in cancer is well-established^6,25–27^. In the present study, KLHL38 was found to promote activation of Akt and its downstream partners by promoting ubiquitin-dependent PTEN degradation. A previous study on KLHL18, another member of the KLHL family, demonstrated that the BTB domains in KLHL18 are critical for the ubiquitination of PI3Kp85α^23^. However, it was not possible here to identify which KLHL38 domains are involved in the PTEN ubiquitination process. Thus, further studies are warranted. PTEN activity is lost by mutation, deletion, or promoter methylation silencing at high frequency in many primary and metastatic human cancers^6^. A549 has a higher number of methylated CpG sites in PTEN^28^. Increased PTEN ubiquitination induced by KLHL38 may also be involved. Moreover, the relationship between KLHL38 and PTEN with respect to the clinical-pathological features of NSCLC remains to be elucidated.

Altogether, these results indicated that KLHL38 promotes the proliferation, migration, and invasion of NSCLC cells, which are correlated with NSCLC clinicopathological parameters. The present data on the survival of NSCLC patients differed from those from the TCGA, possibly because the data were collected from patients with different racial ethnicities. Data from more patients are needed to reduce sampling errors. Collectively, these findings suggest that KLHL38 is a candidate prognostic biomarker and potential therapeutic target for NSCLC.

## Supplementary information

Supplementary Figure Legend

Figure S1

## Data Availability

The results published here are in part based upon data generated by the TCGA Research Network (https://www.cancer.gov/tcga). The data that support the findings of this study are available from the corresponding author upon reasonable request.
