# Supplementary material for: KLHL38 involvement in non-small cell lung cancer progression via activation of the Akt signaling pathway
Source: Cell Death Dis. 2021 May 28;12(6):556. doi: 10.1038/s41419-021-03835-0 (PMC8163838; doi:10.1038/s41419-021-03835-0)
Supplement: Supplementary file 1 — Supplementary Figure Legend [file 41419_2021_3835_MOESM1_ESM.docx]

**Figure S1.** Relationship among PTEN/PI3K/Akt/phospho-Akt pathway. **A**. PTEN, PI3K, Akt and p-Akt levels in A549, and H1299 cells upon downregulation of *PTEN* expression. **B.** PTEN, PI3K, Akt and p-Akt levels in A549, and H1299 cells after treatment with Akt VIII.
