# Supplementary figures and images for: KLHL38 involvement in non-small cell lung cancer progression via activation of the Akt signaling pathway
Source: Cell Death Dis. 2021 May 28;12(6):556. doi: 10.1038/s41419-021-03835-0 (PMC8163838; doi:10.1038/s41419-021-03835-0)

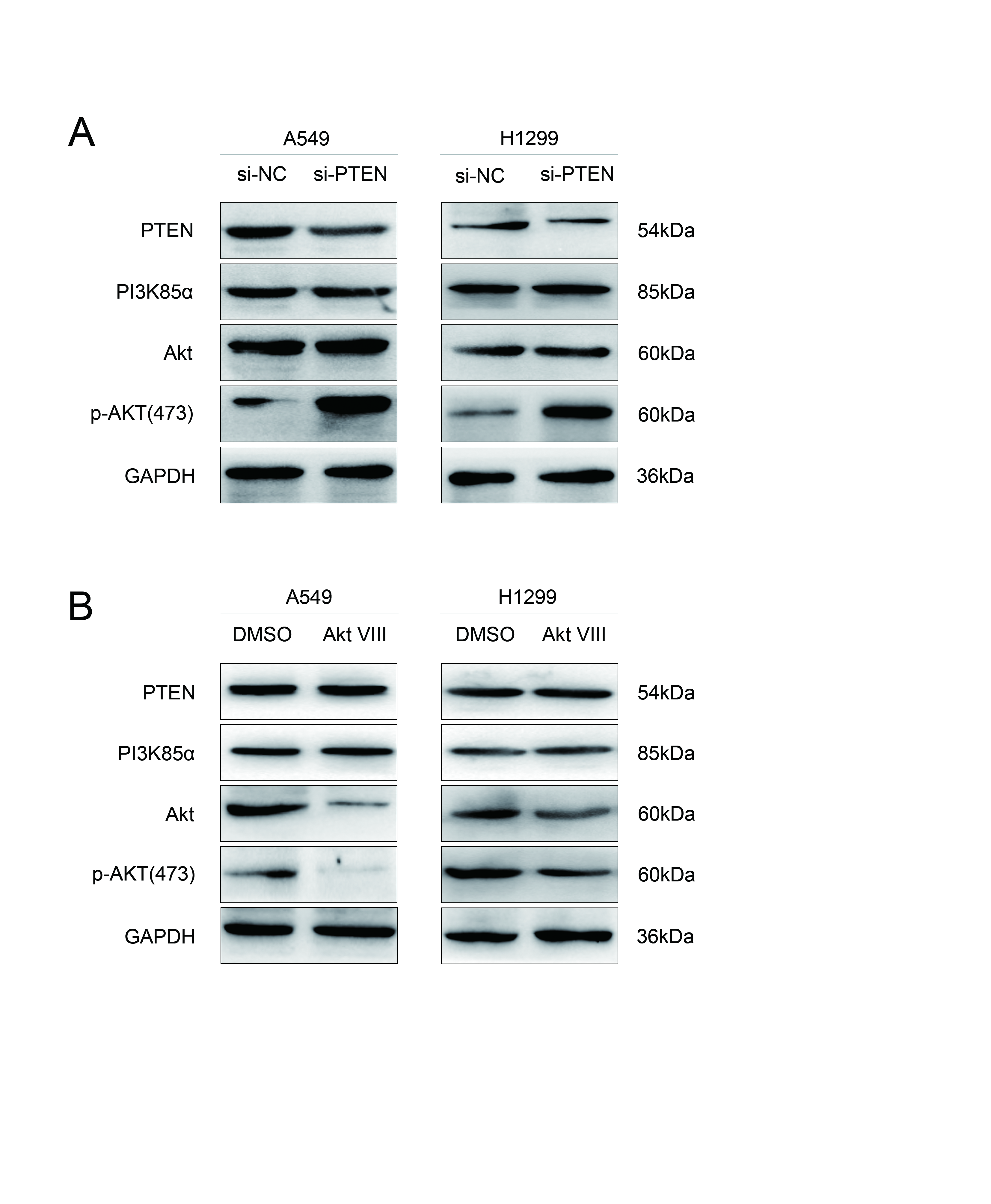

Supplement: Supplementary file 2 — Figure S1 [file 41419_2021_3835_MOESM2_ESM.tif]
